# Effect of Obesity on Lung Function in the Pediatric and Adult Populations with Asthma: A Review

**DOI:** 10.3390/jcm12165385

**Published:** 2023-08-19

**Authors:** Nayely Reyes Noriega, Blanca E. Del-Río-Navarro, Arturo Berber, Sergio de Jesús Romero Tapia, Darío Jorge Mario Molina Díaz

**Affiliations:** 1 Allergy and Immunology Pediatric Department, Hospital Infantil de México Federico Gómez, Ciudad de México 06720, Mexico; naye.rey.nor@gmail.com (N.R.N.); arturoberber@aol.com (A.B.); 2 División Académica de Ciencias de la Salud, Universidad Juárez Autónoma de Tabasco, Villahermosa 86280, Mexico; sergio.romero@ujat.mx; 3 Pediatric Endocrinology Department, Hospital Infantil de México Federico Gómez, Ciudad de México 06720, Mexico; drjorgemariomolina@gmail.com

**Keywords:** lung function, obesity, asthma, pediatric population

## Abstract

Obesity and asthma are major global health concerns, particularly in industrialized nations. Obesity has been shown to have detrimental effects on the respiratory system and lung function owing to metabolic issues and immunological consequences. Research has indicated that obese patients with asthma (atopic or T2-high and non-atopic or T2-low) have diminished lung function in terms of functional residual capacity (FRC), residual volume (RV), expiratory reserve volume (ERV), the FEV1/FVC ratio, and FEF 25–75% due to mechanical fat loading on the diaphragm and central adiposity when compared to non-obese asthmatic patients. Therefore, it is plausible that changes in lung function are the result of a combination of mechanical (fat loading on the diaphragm, central adiposity, bronchial hyper-reactivity, and an increase in cholinergic tone), environmental (diet and exercise), and inflammatory factors (local and systemic), which can lead to the obesity-related asthma phenotype characterized by severe asthma symptoms, poor response to corticosteroid treatment, loss of lung function, and poor quality of life from an early age.

## 1. Introduction

Asthma and obesity are chronic, non-transmittable diseases that are widespread in industrialized countries [1]. According to the National Health and Nutrition Examination Survey 2017–March 2020 Prepandemic Data Files Development of Files and Prevalence Estimates for Selected Health Outcomes, between 2017 and 2020, the prevalence of obesity was higher than 20% among children and adolescents, and 40% among adults [2]. This condition is characterized by excessive fat accumulation caused by a person’s genetic inclination towards overeating from an early age, along with the distribution of fatty tissue (which varies depending on the reference population) and the individual’s interaction with environmental factors such as the availability of food, healthcare services, and family income [1]. Obesity leads to changes in multiple organs and systems, including the respiratory-system-originating alterations in lung volume, augmented resistance, and gas exchange at the alveolar level [3].

On the other hand, asthma is a heterogeneous disorder characterized by persistent airway inflammation and symptoms such as breathlessness, coughing, chest tightness, and wheezing, which can range in severity and expiratory flow limitation [4]. The Global Asthma Network Phase 1 study reported that the worldwide prevalence of asthma in 6–7-year-old schoolchildren and 13–14-year-old adolescents was 9.0% and 11%, respectively, with the highest prevalence of asthma symptoms reported in low- to middle-income countries [5]. Similarly, in 2019, the estimated prevalence of asthma in adults was 9.8%, with the African region being most affected [6].

As the severity of this disease is related to the age of symptom onset, patient phenotype, and the presence of metabolic comorbidities, the purpose of this review was to update and describe the clinical effects of obesity and its repercussions on lung function in asthma patients, providing a clinical and practical overview for physicians.

## 2. The Obesity-Related Asthma Phenotypes

It is generally accepted that obesity can increase the risk of asthma through a variety of mechanisms and is connected to both extrinsic and intrinsic factors that can be linked to the onset of this condition. For example, studies have revealed that maternal obesity and weight gain during pregnancy are linked to an increased probability of asthma symptoms during school age (OR = 1.31: 95% confidence interval [CI] 1.16–1.49) as well as a heightened likelihood of developing obesity during childhood and school age [7,8]. Also, during adolescence, it has been suggested that the effect of obesity on the likelihood of developing asthma is affected by sex hormones, particularly in females with obesity and early menarche [9]. Asthma symptoms in these patients are more intense during the perimenstrual period (also known as perimenstrual asthma) and can result in severe or difficult-to-manage asthma [10] due to decreased progesterone and testosterone levels, as well as fluctuations in estradiol, luteinizing hormone (LH), and follicle-stimulating hormone (FSH) levels, which can trigger mast cell degranulation and result in a heightened respiratory response to aeroallergens [11,12,13].

Chronic inflammation associated with obesity and the early accumulation of central adipose tissue can result in alterations in respiratory mechanics [14,15] and may be further exacerbated by other metabolic conditions such as hyperlipidemia, hyperinsulinemia, and hypercholesterolemia from an early age [16,17]. This persistent inflammation causes changes in the large and small airways, leading to epithelial cell apoptosis, fibroblast activation, increased collagen deposition, thickening of the basement membrane, and increased hypertrophy of bronchial smooth muscle cells, resulting in airway remodeling [18,19]. These histological changes are influenced by different inflammatory pathways based on the patient’s asthma phenotype, which can be identified based on a record of atopy, pulmonary symptoms, age and time of symptom onset, body mass index (BMI), Immunoglobin E serum levels (IgE), cellularity in sputum, the degree of sensitivity to aeroallergens through skin tests, the severity of airway obstruction, and the level of fractional exhaled nitric oxide (FeNO) [20,21]. As a result, asthma patients with obesity can be classified into two subclusters: early-onset (atopic or T2-high) and late-onset (non-atopic or T2-low) phenotypes [22,23,24].

The early-onset or atopic phenotype is characterized by an eosinophilic inflammatory response, with high allergen sensitization since childhood being the most frequent. In addition to the inflammatory markers of the TH2 response, such as interleukin-4 (IL-4), IL-5, and IL-13, high levels of IL-6 and adipokines (such as leptin) due to obesity may have a synergistic effect on airway inflammation and remodeling by stimulating the accumulation of eosinophils, mast cells, and CD4+ T lymphocytes in the airway [25,26]. In contrast, the late-onset phenotype (which is strongly linked to the low-grade systemic inflammation state of obesity also called “meta-inflammation”) is seen in 10–20% of cases and mainly in female patients [27,28]. It is characterized by reduced TH2 markers, neutrophilic airway inflammation induced by type 3 innate lymphoid cells, and airway hyper-responsiveness due to premature closure caused by adipose tissue mediators and TH17 inflammatory cytokines such as IL-17, IL-6, IL-21, IL-22, and Interferon-gamma (IFN-γ). As a result, Tidal volume (TV) is mechanically restricted, and weight gain can exacerbate asthma symptoms and decrease the response to inhaled corticosteroids (ICS) and long-acting beta agonist (LABA) combination therapy [29,30]. For this reason, other treatment alternatives, such as leukotriene modifiers, nonsurgical weight loss strategies, and bariatric surgery, have been investigated with contrasting results [29,30,31,32,33,34]. For example, adult patients with eosinophilic asthma, high levels of IgE, and obesity who underwent bariatric surgery have reported improvements in their lung function (forced expiratory volume in 1 (FEV1) and forced vital capacity (FVC)) one year after the surgical intervention [35,36], control of asthma symptoms or remission [35], and decreased use of inhaled medications compared to patients with late-onset asthma or with normal serum IgE levels [35,36,37,38]. As for children, some studies have reported that a low-calorie diet, exercise, and counseling can reduce exercise-induced bronchospasm with a significant reduction in post-exercise FEV1 drop [39] and can improve FEV1/FVC, reduce asthma symptoms, and increase adiponectin levels [40], suggesting that the asthma phenotype and obesity onset are key factors for the clinical improvement of patients.

**Figure 1:**
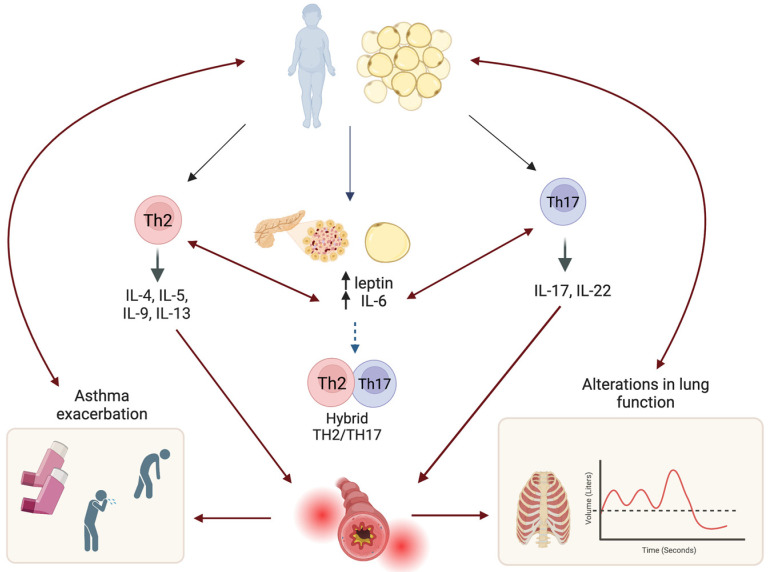
Inflammatory pathways related to asthma phenotypes (Th2, Th17, and Th2/Th17). These pathways can be activated by metabolic alterations, such as hyperinsulinemia and high levels of leptin, due to the meta-inflammation caused by obesity manifesting as severe asthma symptoms and alterations in lung volumes and resistance from an early age. Created with BioRender.com (https://www.biorender.com/, accessed on 17 May 2023).

Interestingly, these phenotypes are not mutually exclusive, and some studies have indicated that the expression of TH17 and TH2 inflammatory mediators is possible, likely because IL17A can be stimulated by IL-13 during allergic inflammation, which then leads to increased expression of TH2 mediators by IL-17, resulting in airway hyper-responsiveness mediated by complement component C5 [41] (See Figure 1).

Another potential modifier of the asthma phenotype is the gut/lung microbiome axis. The microbiome involves the microbiota, metagenome, and surrounding environment that allows microorganisms to interact with different cells, including the immune system [42]. It has already been described that the airway is not sterile, and the presence of *Streptococcus pneumoniae*, *Haemophilus influenzae*, *Moraxella catarrhalis*, and *Haemophilus* spp. since infancy has been associated with the eosinophilic asthma phenotype [43,44]. In contrast, the presence of enriched Proteobacteria increases the expression of TH17 inflammatory pathways, reflecting severe asthma symptoms and suboptimal control [44]. Interestingly, gut dysbiosis has also been associated with asthma since childhood [42]. Recent studies have shown that children with a lower relative abundance of *Faecalibacterium*, *Lachnospira*, *Rothia*, and *Viollenella* (FLVR), slower diversification of the gut microbiome [45,46], and colonization of Clostridium difficile since infancy express higher serum levels of IL-17A, IL-6, and TNF-α, thereby increasing the risk of severe asthma symptoms [45,47]. Consequently, dysbiosis in both the airway and intestinal microbiota could have a synergistic effect on the clinical expression of asthma, as they have the potential to stimulate metabolites (such as short-chain free fatty acids) and pro- or anti-inflammatory cytokines at local and systemic levels, which directly affects the regulation and maturation of mucosal-associated immune tissues (MALT), B-cell differentiation, and dendritic cell (DC) activation, among other immune-critical cells related to protection against pathogens [48]. In addition, there are other external factors that can alter gut and lung microbiota, such as diet, route of birth, breastfeeding, use of antibiotics and antipyretics during the first year of life, the frequency of viral infections, and a history of family atopy, which have been widely described not only as risk factors for asthma but also for obesity [49,50].

## 3. Lung Obstructive Pattern vs. Dysanapsis in Children with Asthma and Obesity

Understanding the impact of inflammation on lung function in patients with asthma is essential for successful treatment and prognosis. In this regard, international guidelines for diagnosing and treating asthma recommend spirometry as the preferred pulmonary study for measuring lung volumes and evaluating the bronchial muscle response to short-acting β2 adrenergic agonists, with FEV1 serving as an indicator of the severity of bronchial obstruction. [4,51]. However, this value relies on the patient’s cooperation and does not always achieve a 12% or 200 milliliter increase after bronchodilator use [4]. Moreover, children with asthma and obesity have a lower FEV1/FVC ratio (a decrease of at least 1% in the FEV1/FVC ratio for every 5 unit increase in body mass index (BMI)) but normal or even increasing FEV1 and FVC, which is in line with either an obstructive pattern or airway dysanapsis [12,52,53,54,55]. Dysanapsis is defined as an imbalance in the growth of the lung parenchyma and airway calibration and is more prevalent in males and in those with obesity from a young age [56]. Even though the mechanisms underlying this phenomenon are still being studied, factors such as weight gain during pregnancy [57], in utero exposure to tobacco [58], and vitamin D deficiency have been associated with it [59,60]. Furthermore, the widespread presence of M1 macrophages and leptin polymorphisms due to excess fat tissue during childhood may impede lung growth and development by stimulating proinflammatory cytokines such as IL-6, IL-12, tumor necrosis factor-alpha (TNF-α), and monocyte chemoattractant protein 1 (MCP-1), which are associated with chronic inflammation and other comorbidities such as insulin resistance or metabolic syndrome, as mentioned later in this review [61,62,63].

A meta-analysis conducted by Forno et al. found that overweight or obese children and adults with asthma had significantly reduced maximum mid-expiratory flow (MMEF of FEF25–75) and a slight decrease in total lung capacity (TLC), residual volume (RV), and functional residual capacity (FRC) [64]. In comparison, children with dysanapsis had a greater FVC, vital capacity, RV, and TLC but a normal RV/TLC ratio, indicating that their lungs were larger with no significant air entrapment [15] (for lung parameter differences, see Table 1). As a clinical resource, it has been observed that the dysanaptic index (FEF50/FVC×Pst(l)50% or FEF25–75/FVC ratio) can be used to predict expiratory flow limitation and response to methacholine, suggesting that dysanapsis may be involved in airway obstruction and hyper-reactivity [65,66]. In addition, patients with dysanapsis are more likely to take three or more asthma medications (odds ratio (OR) 1.72; 95% confidence interval (CI) 1.02–2.90; *p* = 0.04), use short-acting beta 2 agonists daily (OR, 8.3; 95% CI, 1.1–64.0; *p* = 0.04), or experience severe asthma attacks that necessitate hospitalization or systemic steroid treatment (hazard ratio (HR) of 1.40; 95% confidence interval of 1.07 to 1.75; *p*-value of 0.014) [15,55].

## 4. Complementary Studies to Evaluate Lung Function in Patients with Asthma and Obesity

Currently, there are other techniques that evaluate small airway mechanics and monitor pulmonary inflammation, such as the impulse oscillometry system (IOS) and fractional exhaled nitric oxide (FeNO). Both techniques require minimal effort from the patient and are non-invasive [67,68]. While the usefulness of these resources is still being studied for different lung diseases, including chronic obstructive pulmonary disease and bronchiectasis, and in different populations to determine cut-off points due to variability between ethnic groups, age, and comorbidities, various studies have demonstrated their effectiveness in identifying and diagnosing obstructive patterns in the airway and phenotyping patients with asthma [67].

### 4.1. Impulse Oscillometry

The impulse oscillometry system (IOS) is a method that allows the measurement of impedance (Z) and its components, resistance (R) and reactance (X), which are the forces that must be overcome for air to enter and exit the respiratory system to detect alterations in the distal airway by utilizing multiple oscillatory frequencies. In addition, the IOS provides information on the resonant frequency (Fres) and reactance area (AX) [69]. This is useful in patients with alterations in thoracoabdominal compliance, obstructive pulmonary symptoms with normal spirometry, or in obese patients for whom it is not clear whether they have an obstructive or restrictive pulmonary pattern [69,70]. Previous studies have shown a strong correlation between an increase in resistance at 5 Hertz (Hz) minus 20 Hz (R5 − R20) in patients with small airway dysfunction and uncontrolled asthma [71,72]. In addition, the distal obstruction pattern can be identified by the R5 Hz value, which is higher than the upper limit of normality or greater than 1.64 standard deviations (SD) of the predicted value with normal R20 and AX values. According to the Z-value, the degree of obstruction can be classified as mild (1.64 to 2 standard deviations), moderate (≥2 standard deviations), or severe (≥4 standard deviations) [72]. As for IOS bronchodilator responsiveness, it has been suggested that there should be a change of 20–25% to 40% in R5, depending on the cut-off points of each population [72,73,74].

**Figure 2:**
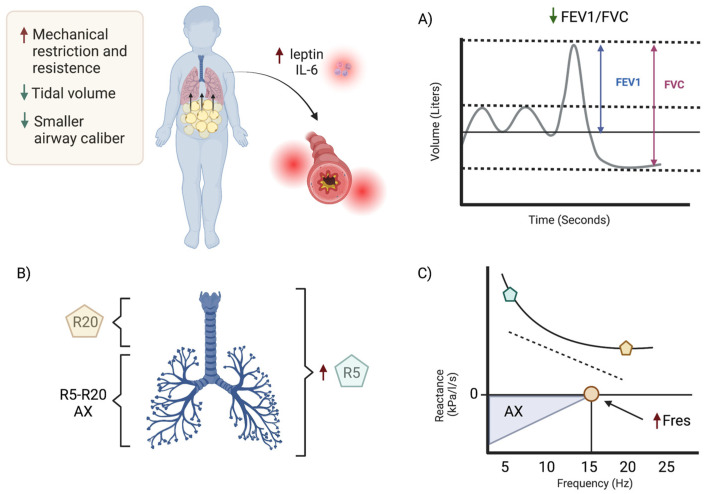
Obesity and proinflammatory metabolic alterations cause changes in respiratory mechanics, promoting a smaller volume and caliber of the airway in patients with asthma. This is reflected in a decrease in the FEV1/FVC ratio (**A**) and an increase in the resistances of both the proximal (R20) and distal airways (R5) (**B**), leading to changes in the reactance area (AX) and resonant frequency (Fres), which is the point where the magnitudes of the capacitive and inertial reactance are equal and have a value of zero at different frequencies (**C**). Created with BioRender.com (https://www.biorender.com/, accessed on 17 May 2023).

However, it has been observed that in children with obesity, the R5, Fres, and AX parameters are higher than those in healthy individuals, and this is reflected in the reference values [75]. The effect of R5 (suggestive of affected small- and large-caliber airways) and the changes in Fres and AX suggest the presence of airway obstruction [75,76,77]. In adults, studies have found that when severe obesity (body mass index (BMI) ≥ 40 kg/m^2^) is present, there is a notable rise in peripheral system resistance (R) and a decrease in reactance (X) [69], and have also established a correlation between the resistance at 5 Hz (R5) and the FEV1 spirometry value (2.5% change in %R5 for every unit in FEV1) [78]. Nevertheless, this association is not always present in children with an obstructive pattern, particularly in those with a history of bronchiolitis or pneumonia during childhood [77]. Consequently, it is recommended that both spirometry and impulse oscillometry be conducted in patients with asthma to define the type and severity of obstruction [79,80,81] (see Figure 2).

### 4.2. Exhaled Fraction of Nitric Oxide

As part of the medical approach for asthma patients, the exhaled fraction of nitric oxide (FeNO) allows for the estimation and monitoring of airway inflammation, which has been relevant in recent years [82]. The inducible or type II isoform of nitric oxide (iNOS) is responsible for the extravasation of macrophages, neutrophils, eosinophils, and T lymphocytes through chemotaxis as well as the expression of proinflammatory cytokines such as TNF-α, IL-1, and IFN-γ, with the consequent overproduction of mucus through the indirect stimulation of TH2 cells in the lungs [83,84]. In relation to the above, an increase in fractional exhaled nitric oxide (FeNO) has been linked to the allergic or eosinophilic asthma phenotype [82,85]. However, patients with asthma and obesity have been found to have low or normal FeNO levels [86,87]. This may have been caused by neutrophils and a low T2-type inflammatory response, which are characteristic of an obesity-related asthma phenotype [88]. Interestingly, some studies in adults have revealed an inverse correlation between FeNO levels and IBM regardless of the presence of asthma [89,90,91,92], and this may be due to the fact that obese patients have narrower airways, which increases the speed of airflow and decreases the time for pulmonary diffusion. Therefore, the amount of nitric oxide in the exhaled air is minimal [93]. Still, further research is needed to determine the usefulness of this measurement in distinguishing between allergic and non-allergic asthma in obese patients.

## 5. Metabolic Alterations Related to Lung Function in Obesity-Related Asthma Phenotype

It is common for individuals with obesity since childhood to display signs of metabolic alterations, such as insulin resistance, elevated levels of leptin, and vitamin D deficiency. In addition to the reduced functional residual capacity due to obesity, these factors can be linked to increased respiratory resistance and changes in lung structure, thereby increasing the risk of bronchial hyper-reactivity and asthma [94].

Research in children and adolescents with metabolic syndrome has shown that both asthmatics and non-asthmatics have decreased FEV1 and FVC values (up to 90 mL) and a reduced FEV1/FVC ratio when insulin resistance, as measured by the Homeostatic Model Assessment Insulin Resistance (HOMA-IR), is elevated, and these changes in lung volume are more pronounced in individuals with obesity and insulin resistance [95,96]. In addition, children with hyperinsulinemia are susceptible to airway dysfunction and heightened airway responsiveness due to epithelial damage and the stimulation of airway smooth muscle growth through insulin growth factor 1 [97,98], which also inhibits neuronal M2 muscarinic receptors and increases the release of acetylcholine from airway parasympathetic nerves, leading to obstructive pulmonary symptoms from an early age [99,100]. These pulmonary changes may also be due to the interaction of other inflammatory agents such as leptin, a hormone that is structurally and functionally similar to IL-6 and directly related to fat tissue [101]. Leptin can activate different signaling pathways that can induce asthma by promoting the proliferation of hybrid TH2/TH17 and TH17-like cells and decreasing T regulatory function by leptin-producing monocytes, resulting in a severe asthma phenotype [101,102,103,104,105,106]. From a clinical standpoint, leptin levels have been observed to be inversely correlated with FEV1 and the FEV1/FVC ratio [107], with a significant decrease during exercise [108] and a poor response to inhaled steroid treatment, which mainly affects adolescent females (see Figure 1) [32,109].

Given the close relationship between obesity, leptin, and insulin resistance, there are various recommendations for the treatment of these conditions with the aim of reducing cardiovascular risk and diabetes mellitus, as well as improving asthma symptoms [110]. Dietary interventions, physical activity, and lifestyle changes have been proposed as the first-choice treatment to promote weight loss and improve insulin sensitivity, respiratory symptoms, and lung function in pediatric patients with asthma. In addition, various studies have reported the beneficial effect of the adjuvant use of pharmacological treatments like metformin (approved by the US Food and Drug Administration in patients older than 10 years) [111], glucagon-like peptide-1 (GLP-1) [112] and dipeptidylpeptidase-4 (DPP4) inhibitors in order to obtain beneficial changes in body weight, insulin, and glycated hemoglobin (HbA1C) [113]. As per a recent study by Foer et al., adult patients with moderate to severe asthma and diabetes mellitus who were treated with glucagon-like peptide-1 receptor agonists (GLP1-RAs) experienced fewer asthma exacerbations over a 6-month period compared to those receiving treatment with insulin, DPP4 inhibitors, or sulfonylureas. The study found that weight loss was a significant contributor to this clinical outcome [114]. Therefore, personalized treatment that takes into account the patient’s age, the severity of obesity, and asthma symptoms is crucial in choosing the best therapeutic option for metabolic alterations.

Vitamin D deficiency has been linked to lung disease due to its role in regulating the immune system by stimulating the expression of anti-inflammatory cytokines such as IL-4, IL-5, IL-10, and IL-13 and inhibiting the expression and transcription of proinflammatory cytokines, particularly IL-17 [115,116]. Additionally, vitamin D plays a critical role in the development of the lung during pregnancy by stimulating type II pneumocytes with receptors for 1,25-(OH)2D3 to increase surfactant production [117,118]. It also suppresses smooth muscle growth and is essential for epithelial–mesenchymal interactions during lung development [119].

Since childhood, low vitamin D levels have been linked to decreased lung function, including lower forced vital capacity (FVC) and forced expiratory volume (FEV1), as well as an increased risk of wheezing, asthma hospitalizations, and an inadequate response to glucocorticoids [120,121,122,123]. These associations may be due to the fact that low vitamin D levels can increase inflammation by elevating the expression of TNF-α and IL-17, while reducing the expression of IL-10, leading to a more intense TH2/TH17 immune response [124]. As a result, studies have been conducted to evaluate the effects of vitamin D supplementation in patients with asthma, and evidence suggests that vitamin D supplementation may improve the response to inhaled corticosteroids in patients with asthma and reduce the frequency of asthma exacerbations [125,126].

Although there are recommendations regarding the supplementation dose in pediatric and adult patients (400–2000 IU/day) [127], there is still no consensus on the appropriate dose to supplement patients with asthma and obesity using high doses of inhaled or oral corticosteroids. This has been the subject of multiple clinical trials (mostly in adults) that have sought to improve the control of asthma symptoms and lung function (specifically FEV1). However, the results have been contrasting because the routes of administration, doses (standard and loading doses), and time of administration are variable, depending on the population and study criteria. A meta-analysis conducted by Lui et al., which analyzed the results of clinical trials of vitamin D supplementation in asthma patients, reported great heterogeneity in the dosage and time of administration of vitamin D, differences in the asthma severity of the included patients, the treatment of asthma at the time of supplementation, and the measurement of clinical outcomes (such as asthma exacerbations, asthma attacks, and control of symptoms using the ACT questionnaire) [128]. Therefore, it is difficult to determine the actual effect of vitamin D supplementation in asthmatic patients with obesity, and more clinical trials with this specific phenotype are required.

## 6. Conclusions

The increasing number of patients with asthma and obesity suggests that the interaction between these two diseases is complex and is influenced by intrinsic and extrinsic factors, which have an impact at the pulmonary level and on the clinical expression of asthma since childhood. Obesity increases the expression of proinflammatory cytokines at the respiratory and systemic levels, which affects the hyper-reactivity of the respiratory tract and conditions the response to drug treatment, which is a challenge for the prevention and treatment of asthmatic patients. Although there are currently diagnostic tools for the evaluation of lung function and asthma phenotyping as diagnostic support, obesity is a comorbidity that must be addressed to improve the quality of life and evolution of asthma. Therefore, patients must be evaluated in an interdisciplinary way to choose the best therapeutic strategy that can modify the progression of both diseases and improve lung function.

## Figures and Tables

**Table 1:** Differences between obstructive and dysanapsis patterns in children with obesity.

Lung Parameters	Obstructive Pattern	Dysanapsis Pattern
FEV1/FVC	↓	↓
FEV1	NL or ↑	NL or ↑
FVC	NL or ↑	NL or ↑↑
FEF 25–75%	↓↓	↓
MEF50	↓	↓
MEF75%	NI	↓
RV/TLC	↓	NL
TLC	↓	↑

Forced expiratory volume in 1 s/forced vital capacity index (FEV1/FVC); forced expiratory volume in 1 s (FEV1); forced vital capacity (FVC); maximum mid-expiratory flow (MMEF or FEF 25–75%); maximal expiratory flow at 50% of the forced vital capacity (MEF50); maximal expiratory flow at 75% of the forced vital capacity (MEF75%); residual volume (RV); total lung capacity (TLC); NL: normal; NI: no information; ↑: increased; ↓: reduced.

## Data Availability

Not applicable.
